# Enhancing the Bioavailability of Resveratrol: Combine It, Derivatize It, or Encapsulate It?

**DOI:** 10.3390/pharmaceutics16040569

**Published:** 2024-04-22

**Authors:** Mohamed Salla, Nadine Karaki, Belal El Kaderi, Abeer J. Ayoub, Samar Younes, Maya N. Abou Chahla, Shairaz Baksh, Sami El Khatib

**Affiliations:** 1 Department of Biological and Chemical Sciences, School of Arts and Sciences, Lebanese International University, Khiyara—West Bekaa, Bayrut P.O. Box 146404, Lebanon; nadine.karaki@liu.edu.lb (N.K.); belal.kadri@liu.edu.lb (B.E.K.); abeer.ayoub@liu.edu.lb (A.J.A.); maya.aboushahla@liu.edu.lb (M.N.A.C.); sami.khatib@liu.edu.lb (S.E.K.); 2 Department of Biochemistry, Faculty of Medicine & Dentistry, University of Alberta, 113 Street 87 Avenue, Edmonton, AB T6G 2E1, Canada; 3 Department of Chemistry and Biochemistry, Faculty of Arts and Sciences, Lebanese University, Zahlé 1801, Lebanon; 4 Department of Biomedical Sciences, School of Pharmacy, Lebanese International University, Khiyara—West Bekaa, Bayrut P.O. Box 146404, Lebanon; samar.younes@liu.edu.lb; 5 INSPECT-LB (National Institute of Public Health, Clinical Epidemiology and Toxicology-Lebanon (INSPECT-LB)), Beirut 1103, Lebanon; 6 BioImmuno Designs, 4747 154 Avenue, Edmonton, AB T5Y 0C2, Canada; sbakshbid@gmail.com; 7 Bio-Stream Diagnostics, 2011 94 Street, Edmonton, AB T6H 1N1, Canada; 8 Department of Biomedical Sciences, School of Arts and Sciences, Lebanese International University, Khiyara—West Bekaa, Bayrut P.O. Box 146404, Lebanon; 9 Center for Applied Mathematics and Bioinformatics (CAMB), Gulf University for Science and Technology, Mubarak Al-Abdullah 32093, Kuwait

**Keywords:** resveratrol, bioavailability, nanoparticle, derivatization, tumor, inflammation

## Abstract

Overcoming the limited bioavailability and extensive metabolism of effective in vitro drugs remains a challenge that limits the translation of promising drugs into clinical trials. Resveratrol, despite its well-reported therapeutic benefits, is not metabolically stable and thus has not been utilized as an effective clinical drug. This is because it needs to be consumed in large amounts to overcome the burdens of bioavailability and conversion into less effective metabolites. Herein, we summarize the more relevant approaches to modify resveratrol, aiming to increase its biological and therapeutic efficacy. We discuss combination therapies, derivatization, and the use of resveratrol nanoparticles. Interestingly, the combination of resveratrol with established chemotherapeutic drugs has shown promising therapeutic effects on colon cancer (with oxaliplatin), liver cancer (with cisplatin, 5-FU), and gastric cancer (with doxorubicin). On the other hand, derivatizing resveratrol, including hydroxylation, amination, amidation, imidation, methoxylation, prenylation, halogenation, glycosylation, and oligomerization, differentially modifies its bioavailability and could be used for preferential therapeutic outcomes. Moreover, the encapsulation of resveratrol allows its trapping within different forms of shells for targeted therapy. Depending on the nanoparticle used, it can enhance its solubility and absorption, increasing its bioavailability and efficacy. These include polymers, metals, solid lipids, and other nanoparticles that have shown promising preclinical results, adding more “hype” to the research on resveratrol. This review provides a platform to compare the different approaches to allow directed research into better treatment options with resveratrol.

## 1. Structure and Therapeutics of Resveratrol

### 1.1. General Structure

**Figure 1:**
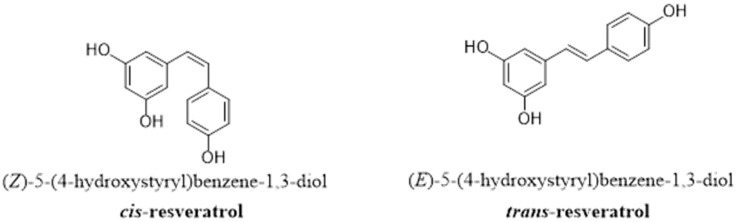
The general chemical structures of both the cis and trans isomers of RSV along with their IUPAC nomenclature.

Resveratrol (hereafter RSV) is a well-known biologically active compound that exhibits a wide spectrum of properties, including antioxidant, cardioprotective, neuroprotective, anti-inflammatory, and anticancer effects [1,2,3]. Structurally, it is a lipophilic polyphenol stilbene synthesized from tyrosine with the action of tyrosine ammonia lyase (deamination), 4-hydroxycinnamoyl-CoA ligase, and stilbene synthase (condensation with three molecules of malonyl-CoA). The molecular structure of stilbene is composed of a 14 carbon backbone and two phenyl rings that are linked by an ethene double bond [4,5,6], and it has a simple structure with a molecular weight of 228.247 g/mol [7,8]. The presence of styrene double bonds allows RSV to exist in two isomeric forms, *cis*- and *trans*-RSV (Figure 1), where the *trans*-form is more common in terms of biological activities and pharmaceutical applications [1,8,9].

### 1.2. Therapeutic Properties

Accordingly, RSV has received significant attention as a promising agent for treating and preventing various chronic diseases, such as neurodegenerative, respiratory, and cardiovascular diseases, as well as diabetes, arthritis, obesity, skin disorders, malignancies, and aging [3,10]. Among the diverse effects of RSV, its ability to act as an antioxidant is the best described [11]. It neutralizes and inhibits reactive oxygen species (ROS), inhibits lipid peroxidation, and chelates metal cations [10,12]. It can be utilized to prevent or minimize lipid oxidation in pharmaceutical products, thus delaying the formation of harmful oxidation products, preserving their nutritional quality and extending their shelf life [13,14]. It has also been effectively used to protect cells against the oxidative stress caused by hydrogen peroxide by promoting cell survival and protecting against cell death induced by UV irradiation [1,15,16]. The synthesis of different esterified acyl chloride derivatives of RSV with better lipophilicity showed that these derivatives may be employed as potential antioxidants in foods and biological systems [17]. Furthermore, studies have demonstrated that the cardioprotective effects of RSV include the inhibition of platelet aggregation [18], the protection of cardiomyocytes from oxidative stress in autophagy, cardiac fibrosis, apoptosis [19], vasodilation [20], antiarrhythmic actions [21], the reduction in the inflammatory state [22], the modulation of lipid metabolism [20], the protection of low-density lipoproteins (LDLs) against oxidative and free radical damage [10], the improvement of left-ventricular function and the reduction in ventricular remodeling in heart failure [23]. Therefore, RSV and its derivatives might be valuable resources for developing novel therapies for the management of heart failure, hypertension, atherosclerosis, ischemia/reperfusion, atrial fibrillation, and metabolic syndrome [21,24].

#### 1.2.1. Neuroprotective Properties

In terms of neuroprotection, RSV has shown protective effects on a variety of neurodegenerative diseases, such as Alzheimer’s disease, Parkinson’s disease, Huntington’s disease, amyotrophic lateral sclerosis, and alcohol-induced neurodegenerative disorders [25,26]. This action is believed to be related to neuronal protection against oxidative damage and toxicity and to prevention of apoptotic neuronal death [27]. RSV is also able to improve acute neurological function and attenuate cerebral edema after intracerebral hemorrhage [28,29]. Furthermore, RSV has a promising role in the prevention and treatment of numerous chronic autoimmune and inflammatory diseases by reducing the secretion and expression of proinflammatory IL-1β, cytokines, TNF-α, and COX-2 and inhibiting the expression of NF-κB and nitric oxide synthase (NOS) [30,31]. For instance, RSV prevented inflammation caused by acute pharyngitis in rabbit models [30]. It also alleviated lipopolysaccharide-induced intestinal and hepatic tissue damage and inflammatory cell infiltration [32]. We have recently shown that several aspirin–RSV derivatives alleviate intestinal inflammation by targeting NF-kB and modulating AMPK [7].

RSV is also effective in treating neurological disorders [33]. It plays a protective role in patients with Alzheimer’s disease [34]. When RSV is combined with donepezil hydrochloride, it improves patients’ inflammatory indicators, facilitates patient prognosis, and promotes patients’ cognitive function [35]. Two different doses of RSV per day, 1000 mg/day and 300 mg/day, in a clinical trial showed that the 1000 mg/day dose selectively increased the psychomotor processing speed from baseline to 90 days posttreatment but had no significant effect on other cognitive functions, such as attention or verbal fluency, across treatment groups [36].

#### 1.2.2. Antitumor Properties and Clinical Trials

The antitumor properties of RSV have been extensively addressed in numerous studies, making it a candidate for the treatment and prevention of different types of malignancies [37]. RSV exerts its anticancer effects via multiple mechanisms [38], such as by inducing autophagy [39] and apoptosis in tumor cells [40,41], inhibiting tumor growth [42] and migration to healthy organs [43] and reprogramming cellular metabolism [40]. It is capable of acting on different protective and common pathways that are generally altered in many cancers, as well as attenuating/inhibiting all stages of carcinogenesis through the inhibition of inflammation, oxidative stress, and proliferation of cancer cells, in addition to the activation of tightly regulated mechanisms of cell death [44,45,46]. For instance, RSV and its derivatives exert anticancer effects on pancreatic cancer cells [47], human cervical carcinoma cells [48], colon cancer cells [49], breast cancer cells [50,51], lung cancer cells [52], and neuroblastoma cells [53], among others.

RSV efficacy has been addressed through more than 271 clinical trials [54] performed globally using different or similar criteria, either combined with another drug or administered alone (summarized in Table 1). RSV can also act as a cardiotonic enzyme inhibitor for dermal health, for platelet aggregation, and for weight management [55] in addition to promoting analgesic effects [56]. The efficacy of RSV depends on several factors, making it more suitable for treating some diseases rather than being a universal drug [33]. For instance, in cancers such as breast, colorectal, lung and prostate cancer, insulin-like growth factor (IGF-1) and IGF binding protein (IGFBP-3) are involved in malignant development. After treatment with RSV for four weeks, a small but significant decrease in IGF-1 and IGFBP-3 was observed compared to the baseline [34]. The potential of RSV to suppress carcinogenic metabolic activity and detoxification by modulating an enzyme involved in cancer progression and transformation results in alterations in cell proliferation and protection against DNA damage in humans [34].

RSV separately or combined with other drugs (such as metformin) can combat cancer. One study has shown that RSV can help in the treatment of glioblastoma (malignant brain tumor) by activating the mTOR signaling pathway, increasing AMPK phosphorylation, affecting different cascades in the MAPK pathway and down-regulating the PI3K/Akt pathway, thus leading to higher apoptosis levels and lower proliferation of cancerous cells reducing tumor volume [57]. Moreover, other studies on RSV also revealed that its anti-cancer effect can be due to the downregulation of circular RNAs, long non-coding RNAs and micro-non-coding RNAs (epigenetic modifications) [58]. Furthermore, the anti-cancer properties of RSV against colorectal cancer have been proven through many clinical trials. In one explanation, RSV acts as a chemosensitizer that modifies many subcellular pathways (p38-MAPK, Akt/STAT3, IL-6/JAK, and other signaling pathways) altering angiogenesis, apoptosis, metastasis, and transcription factors (RSV inhibits the NF-kB and its promoted end-proteins) suppressing cancer plasticity [59,60]. In lung cancer as well, signaling pathways involved in cancer progression such as the mTOR, PI3K/Akt, Wnt/B-catenin are targeted by RSV as a mechanism to reduce the cancer burden [61]. Moreover, RSV protects the liver against hepatocellular carcinoma and induces cancer cell death in cultured cells of hepatocytes in hepatocellular carcinoma by regulating Fas and Fas-ligand. It also inhibits the PI3K/Akt/mTOR pathway inducing apoptosis. In liver cancer, RSV has an anti-angiogenesis effect by blocking the vascular endothelial growth factor (VEGF). Additionally, hepatoma cell proliferation can be inhibited at 100–200 μM of RSV and hepatoma cell invasion suppression at 25 μM of RSV [62,63].

**Table 1:** Summary of clinical trials associated with RSV.

Cancer Type	Study Design/Conditions	Major Findings	Reference
Malignant hepatic tissue (Primary CRC)	▪ 14 days ▪ 5 g of micronized RSV	▪ Increase in the cleaved caspase-3 concentration	[63]
Hepato-cellular Carcinoma and Metastasis	▪ Increasing doses of RSV, ranging from 4 to 32 µM/L to 40 µM/L	▪ Inhibition of the PI3K/Akt/mTOR pathway ▪ Inhibition of NF-κB and induction of apoptosis ▪ Regulation of Fas and FasL levels	[63]
Colorectal cancer	▪ 8 days prior to surgery ▪ 20 patients ▪ 500 or 1000 mg of RSV	▪ Reduction in tumor cell proliferation, indicated by reduction in Ki-67 staining	[64]
Colorectal cancer	▪ 14 days prior to surgery ▪ 9 patients randomized, placebo- controlled, double blind, phase 1 trial ▪ 5.0 g SRT501 (RSV)	▪ Increase in cleaved Caspase-3 (apoptosis)	[65]
Colorectal cancer	▪ 2 weeks ▪ 8 patients phase I pilot clinical trial ▪ 20/80/160 mg of RSV	▪ Non-significant inhibition of the Wnt pathway in malignant tissue	[66]
Breast Cancer	▪ 12 weeks ▪ 34 postmenopausal women with high BMI ▪ 1 gm/day	▪ 10% increase in the concentration of sex steroid hormone binding globulin (SHBG) ▪ 73% increase in urinary 2-hydroxyestrone (2-OHE_1_) ▪ Modulation of hormone-related breast cancer risk factors	[67]
Breast cancer	▪ 6 days ▪ 19 patients ▪ 161.55 mg of RSV	▪ RSV (and its metabolites) concentration in malignant tumors more than in normal tissues	[68]
Breast Cancer	▪ 3 month clinical trial ▪ 39 patients, randomized, double-blind, placebo-controlled clinical trial ▪ 5 or 50 mg of RSV twice daily	▪ Decrease in RASSF-1α methylation	[69]
Prostate Cancer	▪ 4 months ▪ 66 patients, randomized, placebo-controlled, single-site clinical trial ▪ 150 mg or 1000 mg of RSV daily	▪ Decrease in androstenedione, dehydroepiandrosterone (DHEA), and dehydroepiandrosterone sulfate (DHEAS). No effect on prostate size and prostate-specific antigen (PSA) levels	[70]
Prostate Cancer	▪ 2–31 months (depending on patient) ▪ 14 patients, phase 1 trial ▪ 500, 1000, 2000, 3000, or 4000 mg of MPX (every 500 mg MPX has 4.4 μg resveratrol)	▪ Increase in prostate-specific antigen doubling time (PSADT)	[71]
Prostate Cancer	▪ 12 weeks ▪ 22 men, double-blind, randomized, placebo-controlled parallel trial ▪ 30 mg (120 mg/day) in combination with other phytocompounds	▪ Feasible in men with biochemically recurrent prostate cancer and a moderate PSA rise rate	[72]
Multiple myeloma	▪ 4 month study ▪ 24 patients, phase 2 trial ▪ 5.0 g of micronized RSV	▪ No change in biomarkers	[73]

#### 1.2.3. Antimicrobial and Antiaging Properties

Additionally, RSV has been evaluated for its potential to inhibit the growth of some pathogens, such as Gram-positive and Gram-negative bacteria and fungi [74]. It has been found to be a potential beneficial agent for the treatment of *Staphylococcus aureus* pneumonia and other infections induced by *S. aureus* [75]. Pterostilbene, a methoxylated derivative of RSV, has demonstrated antibacterial activity against drug-resistant *S. aureus* [76]. In addition, RSV and dimethoxy RSV derivatives possess antifungal activity against *Candida albicans* [77,78]. It also provides antiviral protection by improving diarrhea induced by rotavirus infection [79] and inhibiting the replication of Pseudorabies virus [80]. RSV is also well known in dermatological applications as a cosmeceutical to enhance skin health as a result of its potential use as a topical anti-aging agent because of its ability to downregulate significant transcription factors implicated in photoaging [81,82].

#### 1.2.4. Cardiovascular Protective Properties

RSV was found to be protective against cardiovascular diseases. Compared with patients in the baseline group, patients with heart failure treated with RSV showed obvious improvement in red blood cell aggregation after three months of treatment, which positively influenced the microcirculation and improved tissue perfusion and the oxygen supply [83]. However, in patients with atherosclerosis, endothelial function, which is a key regulator of vascular homeostasis, is lost. RSV did not seem to improve the cardiometabolic risk factors, sympathetic activity or endothelial dysfunction and did not change endothelium-dependent or endothelium-independent vasodilation [84]. Moreover, RSV also acts as a vasoactive phytoestrogen, affecting cerebrovascular function [85], and can also alter cerebral blood flow variables [34]. Notably, the effect of RSV on patients with migraine was investigated in a three-month randomized, double-blind, placebo-controlled trial, which revealed no worsening or improvement in migraine severity, possibly due to an inadequate duration of treatment or an insufficient/inappropriate dose of RSV [85]. In diabetic patients, RSV was able to reduce insulin resistance, thus improving glycemic control [86], but it had no significant effect on cardiovascular indices or hepatic steatosis in patients with type 2 diabetes [87]. On the other hand, combining RSV with hesperetin (tRES-HESP) improved low-grade inflammation and dysglycemia by reversing insulin resistance and decreasing the expression of CCL2, COX-2, IL-8, and RAGE [88]. In addition, combining RSV with zinc, magnesium, curcumin, selenium, and vitamin D supplements lowered the risk of cardiovascular disease development in overweight and obese children, thus decreasing the possibility of type 2 diabetes, insulin resistance and hypertension and significantly increasing flow-mediated dilation. These observations were revealed by improving both Dickey–Fuller (DF) and post occlusive reactive hyperemia (PORH) tests and DF in the heat provocation test [89].

#### 1.2.5. Other Therapeutic Properties

As an “add on” to meloxicam, RSV has shown efficacy against pain and other symptoms caused by osteoarthritis, which is a painful inflammatory form of joint disease [56]. However, there is still a weak correlation between serum biomarkers and clinical outcomes in patients with knee osteoarthritis [90]. As a phytoestrogen, RSV combined with calcium and vitamin D has a bone-protective effect on postmenopausal women, especially on bones at increased risk of fracture, such as those of the lumbar spine and femoral neck [91].

In addition, negative symptoms such as verbal output, social communication, and loss of emotional and facial expressions developed in patients with schizophrenia and were found to be reduced when RSV was combined with risperidone in another study [92]. Additionally, RSV has been shown to have some beneficial effects on patients with muscular dystrophies, especially on motor function and muscular growth, and it has been shown to reduce ROS levels by inducing the expression of superoxide dismutase [93].

RSV also acts as an antidepressant and has remarkable health benefits. A clinical trial revealed that abnormalities such as anxiety, depression, and serum ammonia levels, which are complications of cirrhosis, developed in patients with minimal hepatic encephalopathy and were reduced when patients were treated with RSV, resulting in improved quality of life [94].

Patients with periodontitis treated with RSV showed improved symptoms in comparison to those treated with the placebo due to a decrease in inflammatory markers in gingival crevicular fluid (GCF) and serum [95].

However, another clinical trial investigating the efficacy of the combination of 500 mg of RSV and 500 mg of curcumin revealed a beneficial effect on muscle and body mass, fat percentage, body mass index (BMI), basal and final serum levels of triglycerides (VLDL), and decreased levels of circulating ferritin in comparison to those in the group treated with the placebo, but there was no significant difference in thiobarbituric acid reactive substances (TBARS) in either group [96]. In a double-blind randomized placebo-controlled trial, patients who received 500 mg of RSV showed a greater increase in Sirtuin-1, resulting in a significant decrease in histone 3 acetylation at the 56-lysine residue (H3K56ac), a decrease in body fat, and a change in ROS balance in patients with chronic diseases (diabetes mellitus and obesity). The small sample size was a limitation of this study [97].

### 1.3. Limitations of Structure and Efficacy

Despite the health and therapeutic benefits of RSV mentioned previously, its translation from natural sources to being used as a therapeutic drug is limited [98], partly because it is rapidly metabolized into glucuronide and sulfate metabolites [99]. In fact, FDA approval has not been granted for the medical use of resveratrol mainly for the lack of robust clinical evidence to meet the FDA’s stringent standards to ensure safety. The FDA approval process may be further hindered by regulatory challenges and bioavailability concerns. Naturally, the quantity of RSV in a source depends on different factors, such as the species, the environment, and the location; RSV may be stored in the skin or the seeds of fruits, which are not usually consumed. At different consumption levels, it is pharmacologically unfeasible to attain the required dose for a desired biological effect. Moreover, RSV is chemically unstable [100], poorly soluble in water [101] and has low bioavailability (less than 1%) because of its extensive metabolism in the intestine and liver [100,102]. Due to its instability, RSV is susceptible to chemical degradation when exposed to UV light, high temperature, pH changes, and oxidative enzymes. Degradation often involves the isomerization of the trans isomer of RSV, which has relatively high antioxidant and anti-inflammatory activity, to *cis*-RSV, leading to a loss of bioactivity after isomerization [103]. In addition, the poor water solubility of RSV makes it difficult to incorporate it into aqueous-based food products. Moreover, its use as a bioactive agent in the pharmaceutical field has also been limited due to its high dose requirement, poor pharmacokinetics, rapid metabolism and elimination [104,105] and low delivery and targeting efficacy at tumor sites [106].

To overcome these limitations, it is often desirable to isolate RSV from a natural source and then incorporate it into an appropriate delivery system. In the next sections, we will systematically summarize common approaches to overcome the limitations associated with the bioavailability, delivery, and therapeutic efficacy of RSV. We will discuss approaches intended to enhance RSV efficacy through combination treatments with other therapeutics, derivatizing the parent RSV compound and encapsulating RSV for potential use within the medical and food industries. The RSV combinations section will address the more common therapeutics and how RSV modulates their activity. Next, we discuss the more relevant RSV derivatives and what is known regarding their biological efficacy in different treatment modalities. We then provide analysis on the major role of nanoparticles (NPs) in overcoming bioavailability limitations and the efficacy of RSV.

## 2. RSV Modifications

### 2.1. RSV Combinations

Oxaliplatin, a platinum-based chemotherapeutic drug, is widely used to treat different cancers, including colon cancer. It induces apoptosis through binding covalently to DNA, thus preventing replication and transcription [107]. However, long-term treatment with oxaliplatin results in drug resistance. A study conducted by Huang H. showed that inhibiting PGE_2_/EP4 receptor signaling can enhance oxaliplatin efficacy in oxaliplatin-resistant colon cancer cells [108]. PGE_2_, the main product of the enzyme cyclooxygenase-2 (COX-2), is a well-known inflammatory mediator that is upregulated in advanced colon cancers since it plays an important role in inducing cell proliferation [109]. Thus, targeting this inflammatory pathway can enhance the efficacy of drugs for treating inflammation-related cancers. RSV was shown to induce apoptosis in different colon cancer cell lines in parallel with the significantly decreased expression of the COX-2 and prostaglandin receptor [110]. Hence, combination therapy including oxaliplatin and RSV should enhance drug efficacy and reduce any associated toxicity. Furthermore, another study showed that this combinatorial treatment resulted in caspase-3 activation, PARP cleavage, and depolarization of the mitochondrial membrane potential, which are all known steps in programmed cell death [111].

Cisplatin, another platinum-based chemotherapeutic drug, is used to treat different types of cancers, including melanoma and lung and liver cancers [112]. The combination of RSV with cisplatin enhances the apoptotic effect of cisplatin in hepatoma cells [113]. RSV can negatively regulate the expression of the glutamine transporter ASCT2, which plays a critical role in the uptake of glutamine, an important amino acid for cancer cells, as it is converted into glutathione for ROS scavenging purposes. This leads to reduced transport of glutamine into cancer cells, therefore altering the redox state in cells treated with cisplatin and increasing their sensitivity to the chemotherapeutic drug cisplatin. The advantage of this treatment is that RSV increases the sensitivity of cancer cells but not normal liver cells to cisplatin, resulting in less toxicity [113].

5-Fluorouracil (5-FU), a chemotherapeutic antimetabolite, is known as a base analog for both uracil and thymine. Its incorporation into growing RNA or DNA strands during replication or transcription interferes with the synthesis of nucleic acids, DNA and RNA and induces cell cycle arrest and apoptosis [114]. Like all chemotherapeutic drugs, 5-FU can cause intolerable toxicity in the majority of patients; thus, using lower doses or combining 5-FU with other drugs can reduce its toxicity, improve its efficacy, and reduce drug resistance [114]. In a study on colorectal cancer cells, it was demonstrated that combining 5-FU with RSV enhanced the efficacy of 5-FU by inhibiting epithelial–mesenchymal transition and downregulating the NF-κB pathway, which is also an important pathway upregulated during inflammation [115]. In a more recent study on human colorectal cancer cells, it was shown that combining RSV with 5-FU enhanced the telomerase activity and apoptosis by inhibiting the STAT3 and Akt signaling pathways [116]. In a murine model of liver cancer, tumor growth was significantly reduced upon cotreatment with RSV and 5-FU [117].

Gemcitabine (Gem) is also a chemotherapeutic antimetabolite that is widely used for treating patients with pancreatic cancer. Similar to other treatments, the development of resistance is the main reason behind lower drug efficacy [118]. In an in vivo study in nude mice, combining RSV and Gem resulted in a greater reduction in tumor growth [119]. In 2019, a study on human pancreatic cancer cells showed that combining RSV with Gem resensitized the cells to Gem treatment by inhibiting the stemness of these cells [103]. Similarly, another recent study conducted in 2021 on pancreatic cancer cells showed that RSV works in combination with Gem in these cells. The decrease in the expression of the angiogenic factor VEGF-B and the inhibition of the activity of the serine/threonine kinase GSK3β by Gem treatment were enhanced upon the combination of RSV with Gem. Furthermore, the decrease in tumor size in mice was more noticeable after this combined treatment [120].

Doxorubicin is an anthracycline used in cancer treatment. This drug acts on cancer cells by blocking the activity of the enzyme topoisomerase II and intercalating into DNA, hence impairing DNA replication and transcription [121]. Combining doxorubicin with RSV has shown promising results in treating different types of cancers, such as breast, gastric, and bladder cancers [122]. SGC7901 gastric cancer cells acquired resistance to doxorubicin through the activation of Akt, which resulted in the induction of EMT. Combined treatment with RSV and doxorubicin has been shown to have synergistic effects on minimizing tumor growth and inhibiting cell migration via the suppression of EMT triggered by RSV through the control of the PTEN/Akt signaling pathway [123]. In a study on HCT116 colon cancer cells, an increase in the expression of the pro-apoptotic protein Bax was observed after the combination of doxorubicin and RSV. Moreover, the sensitivity of these cells to doxorubicin increased upon treatment with RSV since the latter blocks the p-glycoprotein pump, thus favoring higher intracellular levels of doxorubicin [107].

In addition to its effect on inflammation-related cancers, the efficacy of the chemotherapeutic drugs used in endocrine therapy, especially for breast and prostate cancers, is enhanced by RSV. The combination of raloxifene and RSV increased the expression of p53 and caspases 3 and 8 and increased the Bax expression in estrogen receptor-positive MCF-7 breast cancer cells [124]. Another combinatorial treatment composed of RSV and tamoxifen blocked the cell cycle and activated the p53 and p38/MAPK/CK2 signaling pathways in tamoxifen-resistant MCF-7 cancer cells [125]. Bicalutamide, an androgen receptor antagonist, is commonly used in patients diagnosed with prostate cancer. A study conducted in 2019 revealed that the combination of RSV with bicalutamide or an antagonist of CXCR4 (a chemokine receptor known to be upregulated in several cancers) inhibited the AKT signaling pathway, which resulted in the inhibition of prostate cancer progression [126].

### 2.2. RSV Derivatization

Researchers have attempted to produce RSV derivatives with improved bioavailability and more potent biological activities. The molecular structure of RSV can be modified to synthesize RSV derivatives through hydroxylation, amination/amidation/imination, methylation, prenylation, halogenation, oligomerization, and glycosylation [125]. A summary of the main RSV derivatives is provided in Table 2 along with the most distinctive biological activities, followed by a detailed analysis of the structure and function of these derivatives.

#### 2.2.1. Hydroxylated RSV Derivatives

**Figure 2:**
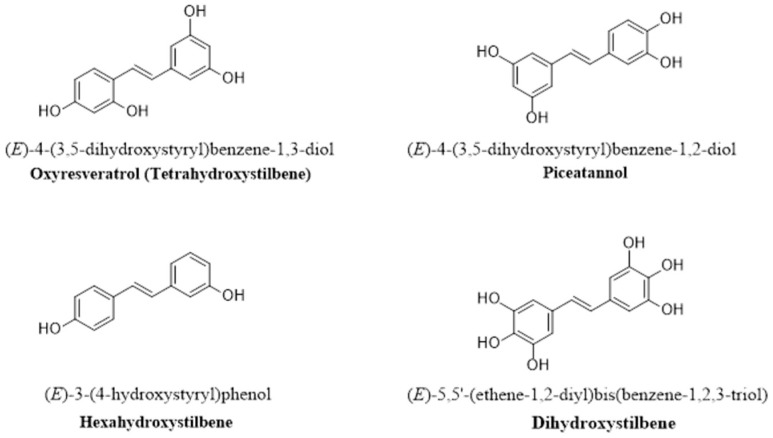
The general chemical structures of several hydroxylated RSV derivatives along with their IUPAC nomenclature.

Adding a hydroxyl group at the 4- and 4′-positions in the *trans* conformation of the stilbenic structure might increase the versatility of the original RSV compound and increase its water solubility. For example, polyhydroxylated RSV derivatives with fewer than three hydroxyl groups exhibit low oral bioavailability [136]. On the other hand, polyhydroxylated derivatives that contain four hydroxyl groups have better water solubility and, in turn, faster absorption, greater bioavailability, and greater metabolic stability than the parent RSV compound [136]. In addition, the number and position of hydroxyl groups increase the metabolic activity of some RSV derivatives. For example, the RSV analogs 3,4-dihydroxtrans-stilbene and 4,4-dihydroxytrans-stilben are more effective antioxidants than the parent RSV compound [137]. Figure 2 shows some of the hydroxylated derivatives that are derived from natural compounds such as oxyRSV (tetrahydroxystilbene), piceatannol, dihydroxystilbene, and hexahydroxystilbene.

Plants that have potential for antioxidant and anti-inflammatory activities are good sources for oxyRSV (*trans*-3,3′,4,5′-tetrahydroxystilbene) [138]. Piceatannol (3′,4′,3,5-tetrahydroxy-*trans*-stilbene), on the other hand, is the most studied hydroxylated RSV derivative and has an additional hydroxyl group compared to that of RSV [139]. Piceatannol has shown to possess antioxidative, anti-inflammatory, anticancer, and immunomodulatory effects [140]. Dihydroxystilbene (4,4′-dihydroxy-*trans*-stilbene) has two hydroxyl groups at the 4′- and 4-positions, which make it more active than the parental RSV component, which has an aqueous solubility barrier that can be resolved by solubilizing it with hydroxypropyl-β-cyclodextrin [137]. Tetrahydroxystilbene (*trans*-3,3′,4,5′-tetrahydroxystilbene) is a natural analog of RSV that possesses stronger tumor-suppressing and antioxidant activity than RSV but is still considered a poorly bioavailable compound [137]. Another synthetic RSV derivative is hexahydroxystilbene (3,3′,4,4′,5,5′-hexahydroxy-*trans*-stilbene), in which additional hydroxy groups improve its biological activity compared to that of the parental RSV compound [137].

#### 2.2.2. Aminated, Iminated, and Amidated RSV Derivatives

**Figure 3:**
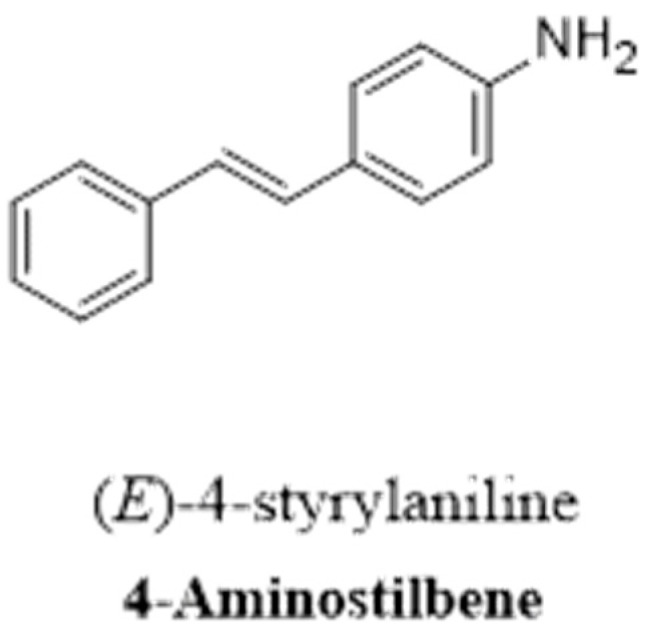
The general chemical structure of aminated RSV derivatives with the IUPAC nomenclature.

RSV can be modified by the addition of amine groups (Figure 3) and the synthesis of amide and imine RSV derivatives. For example, researchers reported the synthesis of aminoalkyl RSV derivatives by cationic peptide [141]. On the other hand, the imine RSV derivative 4-(((2-hydroxyphenyl)imino)methyl)benzene-1,2-diol is a very effective agent that acts as a neuroprotectant [128]. Amidated RSV derivatives are more potent antioxidants than the parent RSV compound. In addition, the allylamine analog *trans*-3,4-dihydroxy-40-(N-allylaminocarbonyl) stilbene showed enhanced protection against glutamate excitotoxicity in neural cells [128].

#### 2.2.3. Methoxylated RSV Derivatives

**Figure 4:**
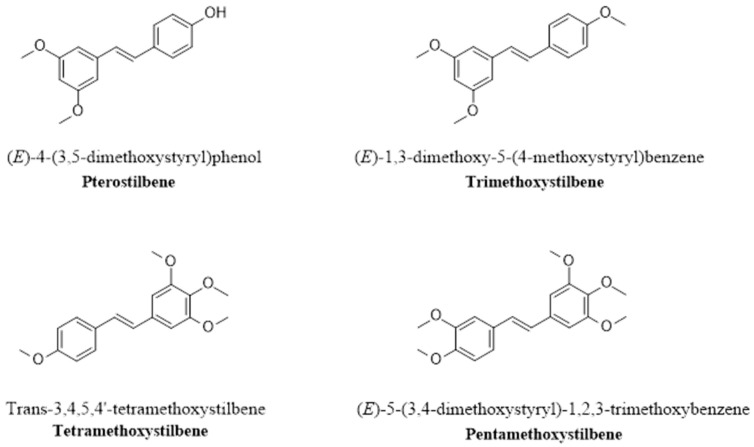
The general chemical structure of methoxylated RSV derivatives along with their IUPAC nomenclature.

The methoxylation reaction involves the addition of a methoxy group to the chemical structure of RSV (Figure 4), which increases its metabolic stability and the time needed to reach the peak plasma concentration [142]. The substitution of a methoxy group with a hydroxyl group in an RSV molecule results in an RSV derivative with increased lipophilicity and increased bioavailability [134,143]. For example, pterostilbene 3,5-dimethoxy-4′-hydroxystilbene is an RSV-derived methoxylated molecule in which two of the three hydroxyl groups are substituted with methoxy groups. This modification increases lipophilicity, improves the oral absorption and cellular uptake of pterostilbene, and increases its metabolic stability and bioavailability compared to those of the parent RSV compound [131]. Trimethoxystilbene *trans*-3,4′5-trimethoxystilbene is another methylated RSV derivative that exists in two isomers, E and Z [134]. Another example of a methoxylated RSV derivative is tetramethoxystilbene *trans*-3,4,5,4′-tetramethoxystilbene, which has more promising pharmacokinetic properties than the parent RSV compound [129] and has shown low toxicity in animal and human models [130].

#### 2.2.4. Prenylated RSV Derivatives

**Figure 5:**
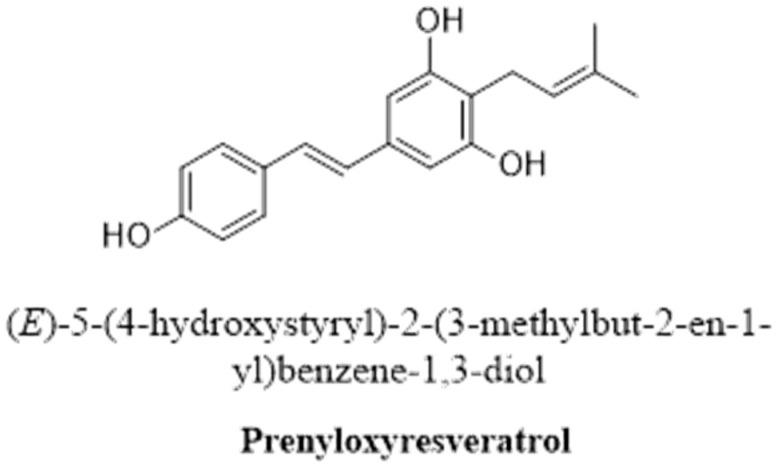
The general chemical structure of prenylated RSV derivatives along with the IUPAC nomenclature.

Prenylation is the covalent binding of a hydrophobic (lipid) moiety to an RSV molecule to produce a prenylated RSV derivative with increased bioactivity (Figure 5) [130]. An example of a prenylated RSV derivative is 3,5,40-trihydroxy-4prenylstilbene, which has shown promising results for the development of drugs [144]. Another example of a prenylated RSV derivative is 5-((E)-2-(3-(3,5-dihydroxy-4-(3-methlbut-2-en-1-yl)phenyl)-2-(4-hydroxyphenyle)-2,3-dihydrobenzofuran-5-yl)vinyl)-2-(3-methylbut-2-en-1-yl)benzene-1,3-diol, which has an increased ability to alter the blood-brain barrier [133].

#### 2.2.5. Halogenated RSV Derivatives

**Figure 6:**
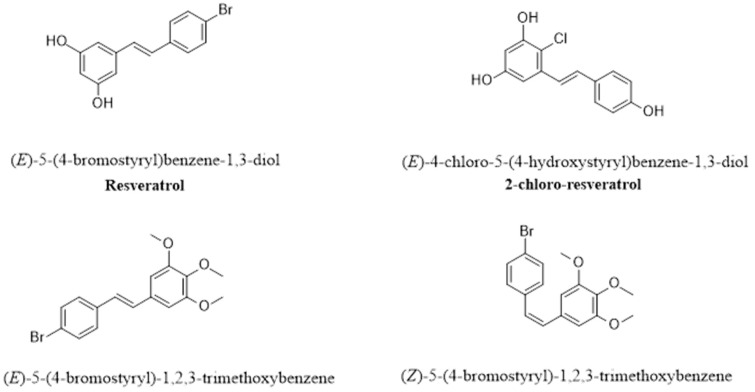
The general chemical structures of halogenated RSV derivatives along with their IUPAC nomenclature.

The addition of a halogen group to RSV might increase its therapeutic potential (Figure 6) [145]. Interestingly, the (E)-2,6-dibromo-4-(3,5-dibromostyryl)phenol RSV derivative showed greater bioavailability than the parent RSV compound. (E)-3,5-Di-fluoro-4′-acetoxystilbene, a fluorinated halogenated RSV derivative, has greater anticancer activity than its parent compound, and 3,4,5-trimethoxy-4-bromo-*cis*-stilbene is more effective at suppressing tumor growth [134]. From an antimicrobial perspective, halogenated RSV derivatives, such as 2-bromo-RSV and 2-chloro-RSV, had lower minimum inhibitory concentrations (MIC) against *C. albicans* than did the parent RSV compound [134], indicating the substantial enhancement of the biological activities attained by halogenated derivatives as compared to the parent resveratrol.

#### 2.2.6. Oligomerized RSV Derivatives

**Figure 7:**
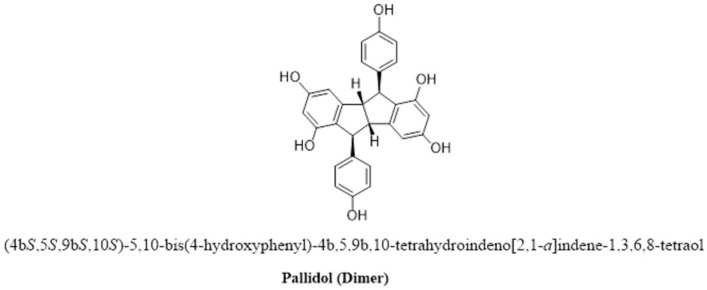

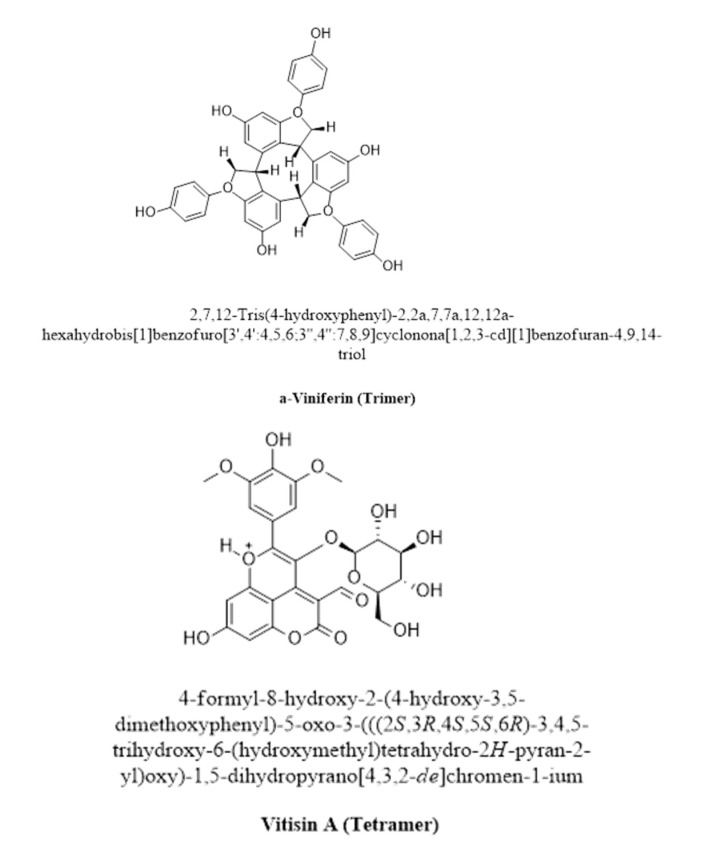
The general chemical structure of oligomeric RSV derivatives along with their IUPAC nomenclature.

RSV oligomerization involves the coupling of monomers via regioselective oxidative coupling (Figure 7) [146]. At least 92 RSV oligomers with biological activities that greatly depend on their molecular size have been reported, indicating that the addition of more RSV units increases the biological effectiveness and specificity of the oligomer. Furthermore, oligomers have a greater scavenging capacity than the parent RSV compound [147].

#### 2.2.7. Glycosylated RSV Derivatives

**Figure 8:**
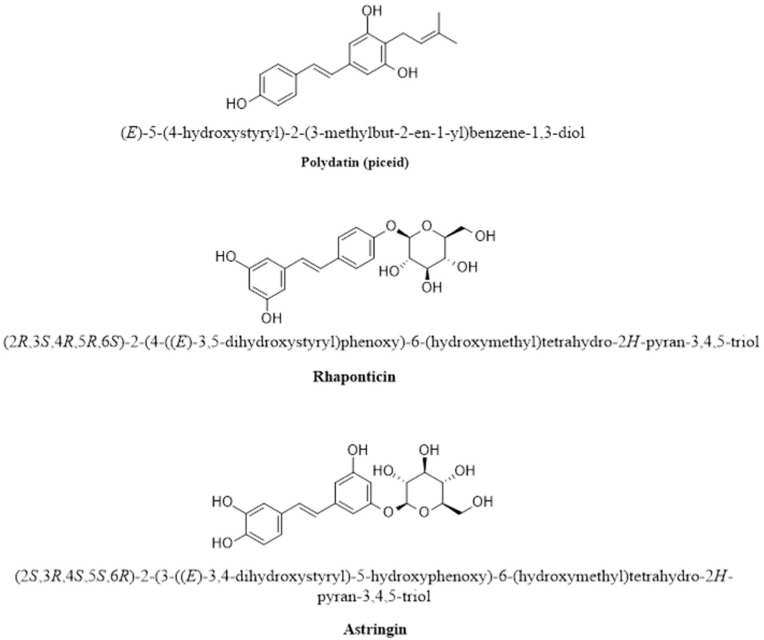
The general chemical structure of glycosylated RSV derivatives along with the IUPAC nomenclature.

Glycosylation is the addition of one or more glycosidic functional groups to an RSV molecule (Figure 8). The addition of glycosidic moieties to an RSV molecule increases its water solubility and bioavailability [148]. An example of a glycosylated RSV derivative is polydatin (also known as piceid) 3,4′,5-trihydroxystilbene-3-O-β-D-glucopyranoside. Polydatin has been reported to have greater bioavailability and less susceptibility to enzymatic oxidation [149]. In addition, polydatin has good oral absorption, antioxidant, and anti-inflammatory properties [150].

### 2.3. RSV Nanoparticles

Researchers have increasingly exploited nanotechnology to encapsulate RSV, aiming to enhance tissue-specific or targeted delivery [151]. Encapsulation is a technique in which RSV, which is the active molecule, is trapped within some form of matrix called the “shell” or the “wall”. Encapsulating RSV improves its water dispersibility, so it can be added to different food products. It also improves its chemical stability, which protects it from environmental factors such as UV light and oxygen. Furthermore, encapsulation can enhance RSV bioavailability by increasing its solubility in gastrointestinal fluids, allowing its absorption by enterocytes and reducing its metabolism before its absorption [103]. There is a large variety of different delivery systems available for the encapsulation of RSV, such as liposomes, niosomes, nanoemulsions, nanoparticles (NPs), and dendrimers [152]. However, in this section, we focus on the different types of NPs that are increasingly used as drug delivery systems to overcome the limitations associated with the use of standard drug formulations. Table 3 summarizes the most relevant nanocarrier systems for RSV and related compounds linked to their biological outcomes.

#### 2.3.1. Polymer Nanoparticles

Polymer NPs are a type of drug delivery system in which the drug is either conjugated or dispersed within the polymer matrix, which protects the drug from degradation, enables continuous release, and enhances its bioavailability. There are various techniques for the preparation of polymer NPs, such as desolvation, dialysis, ionic gelation, nanoprecipitation, solvent evaporation, salting out, spray drying, and supercritical fluid [164]. On the other hand, biopolymer-based systems can be produced using numerous techniques, including extrusion, coacervation, thermodynamic incompatibility, antisolvent precipitation, and emulsion templating [103,165]. However, the choice of an appropriate method depends upon various factors. One of the advantages of polymer-based systems is that they provide high encapsulation and retention efficiency of RSV. In addition, they can be made from natural biomolecules; thus, they are called biopolymers such as proteins and polysaccharides, and they can often be easily made on a laboratory scale. However, problems often arise when scaling up to industrial levels [103]. Several studies have shown that biopolymer nanoparticle-mediated delivery can be a useful approach for improving the bioavailability of RSV. For example, Sarma et al. encapsulated RSV in chitosan–pectin core-shell NPs, which resulted in enhanced RSV bioavailability (almost 30 h) and better in vitro radical scavenging activity than that of free RSV [166]. Sanna et al. developed chitosan-alginate-coated poly(D,L-lactide-co-glycolide) nanoparticles loaded with RSV that provided significant protection against light-induced degradation [167]. Similarly, another study by Detoni et al., showed that encapsulating RSV in NPs provided protection from UV radiation by improving its bioavailability to various targets [168].

Other researchers encapsulated RSV in gelatin NPs, which increased the rate of cellular uptake of the RSV-loaded NPs compared to that of free RES and showed antiproliferative effects on NCI-H460 lung cancer cells [169].

Many studies have shown that the nanoencapsulation of RSV is expected to provide controlled delivery. For example, polymeric NPs encapsulating RSV showed significantly greater cytotoxicity in human prostate cancer than free RSV-induced programmed cell death [170,171]. Furthermore, Zheng et al. (ref) obtained enhanced RSV bioavailability by loading NPs with curcumin and RSV in a successful attempt to target hepatoma cells. These NPs loaded with anticancer drugs reduced the drug dosage and delayed the rate of drug release. They are also highly concentrated in the vicinity of the tumor, which allows them to be considered as potential chemotherapeutic candidates [172].

Protein-based nanoparticles have been used as potential materials for developing delivery systems; nonetheless, there is not a large variety of proteins capable of producing viable nanostructures [173]. Zein is a water-insoluble protein generated from corn. It is used as a coating material, stabilizing polymer and extended-release agent [174]. Zein nanoparticles and modified zein nanoparticles loaded with RSV have been used to enhance the drug loading content (30–57%), improve the antioxidant capacity and stability of RSV [175], and prevent and treat chronic intestinal diseases [176,177]. Moreover, zein-pectin core/shell nanoparticles loaded with RSV have been shown to significantly increase RSV bioavailability and anti-inflammatory activity [178]. The effect of casein-based NPs loaded with RSV on increasing RSV bioavailability to one hundred times greater than that of oral administration of RSV-NPs without casein [179].

Another type of protein-based nanoparticle is silk fibroin (SF). SF is a natural protein polymer with remarkable biocompatibility, biodegradability and low immunogenicity [180]. SF-coated nanoparticles loaded with RSV demonstrated better anti-inflammatory effects and enhanced intestinal barrier function than free RSV in rats with inflammatory bowel disease. The results obtained in rats treated with RSV-loaded NPs revealed decreased expression of inflammatory markers (TNF-α, IL-1β, IL-6, and IL-12) and increased expression of mucins, which are markers of epithelial integrity in the mucosa [181].

Moreover, *beta*-lactoglobin, the major whey protein in bovine milk, is used to prepare protein-based nanoparticles loaded with RSV. These RSV-loaded NPs promoted antioxidant activity against oxidative stress generated by hydrogen peroxide by inducing the cellular uptake of RSV in human lung cancer cells (A549 cells) [182].

Additionally, human serum album (HSA) nanoparticles loaded with RSV were tested in vitro (human liver tumor HepG2 cells) and in vivo (H22 tumor-bearing mice). The coupling of HSA to folic acid (FA) and then encapsulating RSV increased the accumulation of RSV in tumor sites as compared to free HSA encapsulated RSV. A possible explanation could be that HepG2 cells selectively captured FA conjugated HSA and thus increased the uptake of RSV in these NPs. In addition, RSV-loaded HSA NPs combined with folic acid were 5.95-fold more bioavailable than free RSV [183,184,185].

In an in vivo study, RSV-loaded NPs were prepared from poly(ε-caprolactone) and tested against murine melanoma. The results of the present study revealed an increase in the stability of RSV and a significant reduction in the tumor volume in the RSV-treated group compared with the group of mice that received free RSV. Moreover, the nanoencapsulation of RSV led to increased necrosis and inflammation in the cancer area and prevented metastasis [186]. In another study, RSV-loaded polyethylene glycol (PEG)–polylactic acid polymer (PLA) NPs were tested for their in vitro and in vivo anticancer effects. Researchers found that treatment of CT26 colon cancer cells with 40 or 20 μM RSV-NPs for three days significantly reduced the cell number (by 5.6%) and colony-forming capacity (by 6.3%) while enhancing apoptotic cell death and reducing ROS levels. Additionally, when CT26 tumor-bearing mice received 100 mg/kg RSV-NPs intravenously twice per week for three weeks, there was a reduction in tumor growth compared to that in the group receiving free RSV [187].

Few studies have investigated the possibility of RSV delivery into the brain via encapsulation in polymeric NPs. RSV-loaded NPs based on poly(N-vinylpyrrolidone)-b-poly(ε-caprolactone) demonstrated greater neuroprotection than did an equivalent dose of free RSV and a protective effect against ROS in rat cortical cell culture [188]. Moreover, the in vivo encapsulation of RSV in polysorbate 80-coated poly(lactide) NPs also exerted a neuroprotective effect on a neurotoxin that damages dopaminergic neurons and induces Parkinson’s disease-related symptoms in mice [189]. In fact, this polysorbate 80 coating enabled plasma-mediated adsorption of apolipoprotein E (Apo E) to the NP surface, facilitating its recognition by LDL receptors in brain capillary endothelial cells. In addition, compared with free RSV, transferrin-modified PEG-PLA NPs for RSV encapsulation resulted in a high concentration of RSV and a significant reduction in brain tumor volume in glioma-bearing rats [190].

RSV encapsulated in NPs functionalized with phenylboronic acid has been used to accelerate infected wound healing by targeting bacteria and solving the water-insoluble problem of RSV. Compared with free RSV, the RSV NPs inhibited the expression of inflammatory cytokines and reduced the amount of excessive ROS inside the cells. The application of a gel embedded with RSV NPs enhanced the formation of granulation tissue, collagen deposition, and re-epithelialization, smoothing wound healing [191].

#### 2.3.2. Metal Nanoparticles

Metal NPs are multipurpose agents. For example, they can be used in photothermal cancer therapy [192,193]. Metal nanoparticles are synthesized via different methods, such as spray pyrolysis, the colloidal gel technique, chemical vapor deposition, electrodeposition, and rapid solidification processing [194]. The advantage of these nanocarriers is the ability to control drug release depending on environmental factors, such as pH. The delivery performance and anticancer efficacy of RSV were enhanced when RSV was conjugated with gold nanoparticles (Au SNPs) using polyvinylpyrrolidone as a cross-linker. Compared with free RSV, these RSV-loaded NPs reduced the proportion of necrotic cells by activating the mitochondrial intrinsic apoptotic pathway in human pancreatic cancer cells [195]. Other studies on the encapsulation of RSV in AuNPs have shown that RSV induces cell cycle arrest in MCF-7 cancer cell lines [196] and has better anti-invasive effects on human breast cancer cells than RSV alone [197].

In another study, hollow Au NPs coated with RSV were fabricated to improve the photothermal activity and cytotoxicity against melanoma. These NPSs could block the cell cycle, inhibit cell division, and induce apoptosis after 808 nm laser irradiation in A375 melanoma cells. Because these NPs contain no surfactants, surfactant separation procedures and surface modification procedures are not needed for most theragnostic materials [198].

To identify a treatment for diabetic retinopathy, which is an important cause of acquired blindness, Dong et al. developed AuNPs coated with RSV through an ecofriendly synthetic process and tested their effects on rats with induced diabetic retinopathy. RSV was used as both a stabilizing agent and a reducing agent. RSV-loaded AuNPs reduced the permeability of the blood–retinal barrier and reduced retinal inflammation by repressing the nuclear factor NF-kB. This latter process is known to promote the development of exacerbated oxidative stress and proinflammatory cytokines and leads to apoptosis. In conclusion, RSV-coated AuNPs have demonstrated anti-inflammatory properties for the treatment of retinal diseases [199,200].

Moreover, RSV encapsulated in metallic NPs has shown potential for application as a nanoantibacterial agent with improved activity. Park et al. developed RSV-AuNPs and RSV-silver NPs (AgNPs) using green procedures, where RSV was used as a reducing agent to chemically reduce gold and silver ions to AuNPs and AgNPs, and it also played a role as a capping agent in RSV-AuNPs. This study showed that both types of metallic NPs had greater antibacterial activity against gram-positive and gram-negative bacteria than did RSV alone. Among the tested strains, the greatest antibacterial activity of the RSV-AuNPs was observed against *Streptococcus pneumoniae* [201].

#### 2.3.3. Lipid-Based Nanoparticles

##### Nanomicelles

Micelles are self-assembling nano-sized constructs (10–100 nm) with a hydrophobic core and hydrophilic shell. They provide an improved solubility and, therefore, a better intestinal permeability of micelles. Polymeric micelles are formed of amphiphilic block copolymers and can range in size up to 200 nm [202]. They are of important use in tumor targeting due to their prolonged circulation time after systemic injection and their accumulation at tumor sites. Polymeric micelles tend to be more stable than surfactant micelles because they have lower critical micelle concentrations (CMC), slower dissociation rates, and reach a high drug concentration at the target site [203]. Due to its cost-effectiveness and good bioavailability, pluronic F68 is a popular material for the synthesis of micelles. However, its high CMC significantly can decrease the drug encapsulation efficiency, and, thus, it is crucial to balance cost effectiveness and encapsulation abilities for many polymeric nanomicelles. In fact, pluronics alone have insufficient drug encapsulation capacity and high critical micelle concentration (CMC), leading to the dissociation of micelles after intravenous injections. To overcome this, pluronics are often used with other micelle copolymers to increase drug loading capacity. For instance, Gregoriou A et al. showed that the encapsulation of RSV in polymeric micelles (with dominant size of 179 ± 22 nm) using pluronic F127 block copolymer and Vitamin E-TPGS was highly efficient (73 ± 0.9%) and the drug loading content was of 6.2 ± 0.1. These RSV-loaded micelles reduced the viability of breast cancer cells with no effect on the control cells [204].

In addition, Radeva et al. developed double-loaded mixed pluronic micelles with doxorubicin and resveratrol using the film hydration method. The nanomicelles size were less than 200 nm and the loading efficiency for both drugs was high (83.4% for doxorubicin and 78% for resveratrol). In vitro tests of drug release were performed in two different media with pH 7.4 and 5.0. Results showed that in both release media, there was a faster release of doxorubicin compared with resveratrol, and it was even more marked in the acidic than the alkaline medium. The study demonstrated that the simultaneous delivery of doxorubicin and resveratrol in pluronic micelles enhanced the cytotoxicity of doxorubicin in lymphoma cells and lowered its cardiotoxicity in cardiac cells [156]. Yet, another study by Kamenova et al. reported the development of a micellar system based on a poly(methacrylic acid)-b-poly(ε-caprolactone)-b-poly(methacrylic acid) triblock copolymer for the oral delivery of resveratrol. The 100 nm micelles were first formed in an aqueous media via the solvent evaporation method and then loaded with resveratrol (72% encapsulation efficiency). A pH-sensitive release strategy was adopted (pH of 1.2 and 6.8 similar to those of the gastrointestinal tract), and results showed that approximately 86% of RSV was released in the medium resembling the intestines’ pH (6.8), while at the gastric pH (1.2), the released drug was 74%. In addition, the micellar RSV shielded epithelial cells from damage due to inflammatory cytokines which makes it useful in the treatment of inflammatory gastrointestinal diseases [205].

##### Nanoemulsions

Nanoemulsions, lipid-based pharmaceutical systems provide a versatile method for delivering drugs through lipophilic barriers. These systems consist of two immiscible liquids (oil and water); one in the dispersed phase and the other in the continuous phase. These nanoformulations are typically stabilized by emulsifiers such as surfactants and co-surfactants [206]. Usually, the preparation of an emulsion is carried out using high-pressure homogenizers, high shear stirring, or ultrasound generators as external forces to promote the release and absorption of the drug after digestion while improving targeted drug delivery [207]. For example, RSV nanoemulsion-based gels have been developed for the topical delivery of the antioxidant RSV for the prevention of UV-induced oxidative skin damage due to the RSV-enhanced skin permeability and retention effect [208].

RSV-nanoemulsions have been used in the treatment of diseases such as cancer, autoimmune disease, parasitic infections, and others. For instance, methotrexate-RSV loaded nanoemulsions have been developed to surmount the bioavailability problems and the adverse effects of rheumatoid arthritis monotherapy. This nanoformulation loaded with RSV showed 79% inhibition in inflammation and better anti-arthritic effects [209].

In another study, RSV-based oil-in-water nanoemulsions were shown to be effective against bladder cancer. By facilitating rapid intracellular drug uptake, RSV-nanoemulsions reduced the viability of bladder T24 cancer cells and intensified the cytotoxic activity of RSV. These results strongly indicate that the utilization of nanoemulsions can effectively enhance the bioavailability of RSV [210].

Resveratrol nanoemulsions were also used as an anti-leishmanial therapeutic against *Leishmania major*, the parasite that causes leishmaniasis. RSV-loaded nanoemulsions were prepared using the probe ultra-sonication method and tested for anti-leishmanial activity using different concentrations. This study showed that, in comparison to the control group, both RSV and RSV-nanoemulsions at all concentrations demonstrated significant inhibitory effects against leishmania and that both are safe for mammalian cells. However, there were no significant differences between the anti-leishmanial effects of RSV and RSV-nanoemulsions [211].

Furthermore, Kotta et al. prepared coconut oil-based resveratrol nanoemulsion using pluronic-107 and cremphor EL as surfactants. The nanoemulsion exhibited superior drug release properties in comparison to an RES suspension in 0.5% (*w*/*v*) sodium carboxymethyl cellulose, and demonstrated an effective brain-targeting effect upon intranasal administration in rats. Additionally, the nanoemulsion remained stable at room temperature for a period of 3 months [212].

##### Liposomes

Liposomes are traditional models of lipid-based formulations which were invented in 1965. They are defined as stable spherical vesicles made of amphiphilic lipids. They can be easily made in an aqueous environment by controlling the temperature, the pH, and the ionic strength, to allow lipids and phospholipids to assemble into spherical bilayers [213]. There are several methods for liposome preparation such as thin-film hydration, reverse-phase evaporation, and microfluidic mixing [214]. Several studies have used liposomes as a biocompatible and smart delivery system to carry RSV.

Liposomal encapsulation of resveratrol has been shown to improve the solubility, stability, and bioavailability of RSV, enhancing its therapeutic effects, such as the anti-cancer properties [215], antibacterial properties [216], wound healing properties [217], reduction in induced nephrotoxicity [218], among others properties, by protecting it from degradation and facilitating its delivery to targeted cells or tissues.

Zhu et al. synthesized RSV-loaded liposomes modified with folate (FA-RSV-liposomes) in order to evaluate antitumoral activity against the human osteosarcoma cell 143B. According to the findings, the FA-RSV-liposomes increased apoptosis while inhibiting tumor cell growth. Folate-modified liposomes had significant antitumor efficacy in comparison to free RSV [219].

Xu et al. encapsulated RSV in a liposomal nanoparticle of 333 ± 50 nm; the encapsulation efficiency was about 85%. The encapsulated material showed a stronger ability to reduce reactive oxygen species when compared with free RSV which demonstrate their potential ability to deliver poor bioavailable nutrients [220]. 

In addition, Huang et al. used the thin-film hydration technique of egg yolk phosphatidylcholine and Tween 80 to make liposomes loaded with both RSV and curcumin. The liposomes had a diameter ranging from 75 to 90 nm. When compared to their individually loaded counterparts, co-encapsulated liposomes demonstrated greater antioxidant and lipid peroxidation inhibitory characteristics, as well as improved stability [221].

Moreover, Peng et al. studied the use of pH-driven techniques paired with high-pressure homogenization to create polyphenol-loaded liposomes containing curcumin, quercetin, and RSV. Each polyphenol had a distinct encapsulation efficiency depending on its stability in alkaline environments, with the highest for liposome-loaded curcumin at 100% efficiency, RSV at 93% efficiency, and quercetin at only 54% efficiency. Thus, the aforementioned findings demonstrate that adopting pH-driven techniques to encapsulate lipophilic polyphenols is dependent on the impact of pH on the stability and solubility of the bioactive molecules [222].

Cadena et al. showed that encapsulation of quercetin and RSV into elastic liposomes was highly efficient (up to a 97%) and liposomes presented a mean diameter of 149 nm with a polydispersion index of 0.3 which makes them suitable for subcutaneous injection used for reducing subcutaneous fat [223].

Despite the advantages of liposomes, many of their applications are limited due to their high production costs, short shelf life, poor stability, rapid removal by the reticuloendothelial system, and cell interactions.

#### 2.3.4. Solid Lipid Nanoparticles (SLNPs)

SLNPs are another carrier system that combines the benefits of both polymeric NPs and lipid emulsions. The spherical vesicles contain a solid lipid core surrounded by hydrophilic surfaces. Although they can transport both hydrophobic and hydrophilic agents, they are especially useful in the delivery of hydrophobic drugs due to their high affinity for the core [224], so RSV, a hydrophobic drug, can be easily incorporated into the lipid core. The fabrication of SLNPs can be achieved through the hot homogenization method or the solvent emulsification and diffusion technique [225]. The advantage of using SLNPs is their easy uptake by cells, which makes them good nanodevices for the delivery of transdermal drugs [226,227]. For example, RSV-loaded SLNPs cross the phospholipid bilayer in less than 15 min [228]. Additionally, when tested for their potential use in skin disorder therapies, it was shown that RSV-loaded SLNPs were more effective than kojic acid, a melanin inhibitor, at inhibiting tyrosinase and proved to be nontoxic to HaCaT keratinocytes [229]. The same effect was produced by RSV-loaded SLNPs prepared with polyoxyethylene 40 (POE40) stearate lipid. These compounds even showed greater encapsulation efficiency and greater inhibition of tyrosinase than did RSV-loaded SLNPs alone or those prepared with glyceryl behenate, which is more hydrophobic than POE40 stearate [230].

Moreover, these lipid nanocarriers have been shown to be effective RSV delivery systems that could be used to improve the treatment of brain diseases such as glioma. Few studies have reported the delivery of RSV to the brain through the blood–brain barrier when it is loaded in SLNPs. The results showed that RSV-loaded nonfunctionalized SLNPs significantly increased the RSV concentration in the brain compared to that of free RSV but had a lower RSV concentration than that obtained with RSV-loaded nonfunctionalized SLNPs [231,232]. It can be concluded that the functionalization of SLNPs with ApoE indeed improved the permeability of RSV-loaded SLNPs across the BBB and thus increased RSV delivery to the brain.

The ability of RSV-loaded SLNPs to attenuate doxorubicin-induced cardiotoxicity was analyzed. In fact, RSV is known to attenuate the cardiotoxicity of doxorubicin, a chemotherapeutic drug that is clinically used to treat breast and lung cancer. However, the poor water solubility of RSV makes it difficult to achieve a satisfactory effect after oral administration. Thus, its encapsulation in SLNPs was found to improve the bioavailability of RSV to better protect the myocardium and inhibit doxorubicin-induced cardiac toxicity [233].

RSV-loaded SLNPs were also analyzed for the delivery of RSV to human breast cancer cells. An in vitro study on the delivery of RSV to human breast cancer cells using RSV-loaded SLNPs showed that the latter blocked the cell cycle and reduced metastasis to a greater extent than free RSV. In addition, RSV-loaded SLNPs were more effective than free RSV at inducing cell apoptosis, probably because the water solubility of RSV improved when RSV was incorporated into SLNPs [234]. These results suggest that RSV-SLNPs may be potential therapeutic agents for breast cancer treatment.

Another promising tool against cancer is the co-encapsulation of a cytostatic drug such as docetaxel with a natural chemosensitizer such as RSV in epidermal growth factor-conjugated SLNPs. In vitro and in vivo studies have shown that these SLPNs have significant synergistic effects, the greatest extent of tumor inhibition, and the lowest systemic toxicity in comparison to free docetaxel; thus, these SLPNs can be used for the treatment of non-small cell lung cancer [235]. The advantage of the simultaneous administration of docetaxel and RSV is that it can overcome multidrug resistance, which often impairs the efficacy of traditional chemotherapy [236].

#### 2.3.5. Nanostructured Lipid Carriers

Nanostructured lipid carriers (NLCs) are the second generation of SLNPs. They are composed of a mixture of solid lipids and liquid lipids in the nanocore, usually in a ratio of 7:3 to 9:1 and have an average size between 10 and 500 nm [237]. NLCs are thought to be an upgraded version of SLNPs, with the same distinctive features but an optimized core composition, resulting in a higher drug loading capacity, greater stability, and the ability to work at lower temperatures. It is worth noting that NLCs remain solid even at body temperature [238]. Just like SLNPs, NLCs are prepared using the high-pressure homogenization (most commonly used), solvent emulsification/evaporation, supercritical fluid extraction of emulsions, ultrasonication and spray drying [239]. Recent studies have shown that RSV-loaded NLCs prepared using interfacial polymer deposition can improve acute lung injury [240],protect blood vessels, and improve the antihypertensive effects of RSV [241].

NLCs (composed of the lipids trimyristin and triolein) loaded with RSV were prepared via probe sonication. The particle sizes were less than 100 nm, the encapsulation efficiency was as high of 97%, and the drug loading was about 3.4%. The release of RSV from NLCs was retained with the maximal release of RV observed after 12 h (27.7%) which confirms the ability of NLCs to protect the entrapped drug, reducing its release and degradation in the outer media.

This study showed the ability of RSV-loaded NLCs to restore vasodilator responses in an ex vivo model of acute hypertension [241].

NLCs have also demonstrated the capacity to protect the skin by delivering RSV to the epidermis. A nanostructured lipid carrier gel loaded with RSV was prepared in an average particle size of 175.6 ± 11.2 nm and with an RSV encapsulation efficiency of 97.76%. Results showed improved stability of RSV under ultraviolet irradiation and its accumulation in the epidermis. This RSV-loaded NLC gel could scavenge free radicals effectively and protect human keratinocyte from UV irradiation by inhibiting the generation of ROS [242].

**Figure 9:**
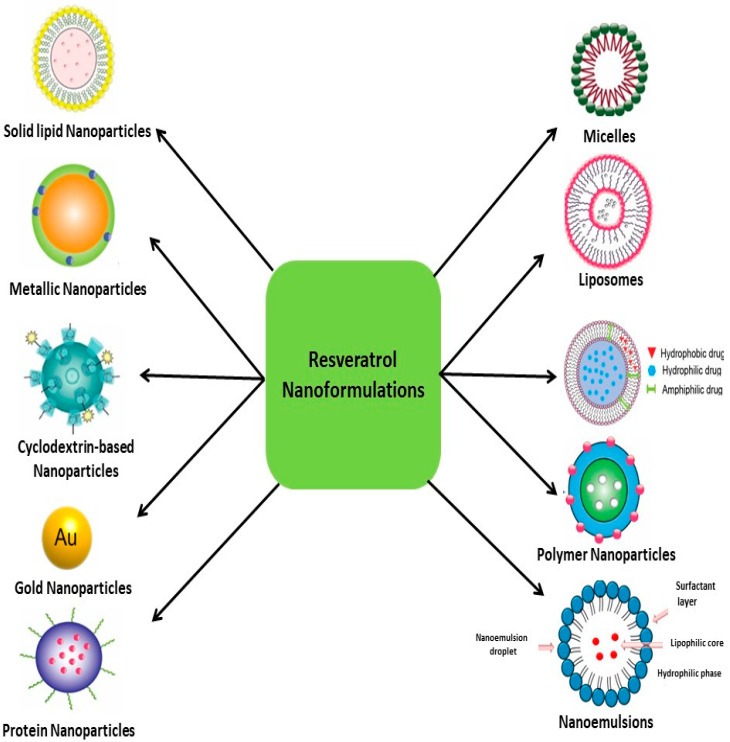
The more common RSV-based nanoformulations.

To conclude, there is no doubt that RSV encapsulated in nanocarriers overcomes the hurdle of physicochemical characteristics and has shown promising results in preclinical studies in terms of its therapeutic efficacy. However, the suitability of RES nanocarriers still needs to be investigated in human trials. In addition to the above-discussed RSV nanoformualtions, Figure 9 brings together the more common RSV-based nanoformulations.

## 3. Discussion and Future Directions

Inflammation and inflammation-associated disorders such as cancer have been treated with natural compounds derived from plants for the longest time. Extensive studies have aimed to investigate the efficacy of natural compounds as anti-inflammatory, antitumor, and other therapeutic agents. Two important concepts are generally connected to RSV: the French paradox and calorie restriction. The first was introduced approximately 30 years ago and describes the epidemiological observation that French people have a relatively low rate of cardiovascular diseases even though their diets are rich in fats. The observation may not be fully scientifically validated, but one implication has gained much interest; a component of the French diet must have a protective role against coronary complications. In fact, it was shown later that RSV protected against plaque formation in different animal models of atherogenesis and enhanced nitric oxide production to improve vasodilatation. This provided a possible partial explanation for the French paradox.

The concept of calorie restriction refers to the moderate intake of food and thus a reduction in calorie intake without leading to malnutrition. It is one of the most reliable nonpharmacological applications that increases life span in model organisms, including rats, insects, fish, and mammals. The link between RSV and caloric restriction effects emerged with the discovery that the protein Sir2 (silent information regulator 2) is involved in the regulation of aging in yeast.

In general, preclinical studies have yielded promising results on the benefits of RSV for the management of a variety of diseases related to inflammation as well as for cancer treatment. It is important to know that the treatment and/or prevention of most diseases in animals do not always translate to human studies, so care should be taken in our interpretation of the effectiveness of RSV in treating humans.

One of the greatest challenges associated with RSV is its low bioavailability. RSV is highly absorbed when given orally, and it has very low bioavailability due to the rapid metabolism of its glucuronide and sulfate conjugates. Although this latter concept may explain the in vivo results observed for RSV and for the treatment of disease in humans, whether appropriate target cells can also transport the sulfated conjugates of RSV into the cell to be metabolized back to the parent compound needs to be investigated.

Due to the poor bioavailability of RSV, it is unclear which dose should be used for clinical studies. This dose is particularly important because several effects of RSV are dose dependent. Furthermore, since RSV may exhibit a hormetic dose-response effect, this further complicates dose selection for clinical studies. Another important and related question is what method of administration should be chosen.

Although RSV is poorly soluble in aqueous solutions, some preclinical studies have administered RSV in drinking water, which may create dose variability issues. How this hurdle will be overcome in clinical trials has yet to be addressed, but the poor solubility of RSV may be enhanced by increasing its aqueous solubility via microparticulate systems, cyclodextrin complexes, nanocarrier systems, or even vesicular systems. However, whether this approach can improve the effectiveness of RSV in clinical studies is yet to be tested. For the aforementioned reasons, the bioavailability of RSV derivatives needs to be fully investigated for clinical use. Our previous results provide in vitro and in vivo insights into these RSV derivatives, but further usefulness will require extensive pharmacokinetic analysis and bioavailability determination before human clinical trials are attempted [7].

In summary, given the valuable health benefits of RSV, novel methods involving the use of “modified” RSV in clinical trials are urgently needed. Combinatorial therapy including polyphenols such as RSV can be a mechanism to lower chemotherapy-associated toxicity. In fact, catechins, which constitute another class of polyphenols, are reportedly used among classical antitumor drugs [243]. Combination treatment including polyphenols are well reported for their synergistic outcomes. RSV, containing a free hydroxyl group, can interact with a plethora of other drugs to enhance their therapeutic ability. However, the reactivity of the hydroxyl group needs to be carefully monitored for potential conjugation with cellular components such as protein and lipids. In fact, RSV has been described in several reports as a hormetic drug, showing protective outcomes at lower doses and more toxic ones at higher doses. This can be further attributed to the fact that the RSV acts as an antioxidant at low doses and a prooxidant at higher doses [244].

The rise of nanoparticles and delivery systems has perhaps reignited the hype around RSV, by enhancing the physiochemical properties. Increasing the half-life of RSV and its association with serum albumin can be promising in ensuring better biodistribution and bioavailability. A key point which remains to be answered in pre-clinical and clinical research with RSV is how long a course of RSV treatment should be. As noted in Table 1, the time frame of treatment for most existing clinical trials is quite different. An interesting speculation would be whether a low dose (antioxidant) and long-term supplementation of RSV will be tolerable in humans. In fact, an interesting observation in middle-aged primates fed on RSV for 2 years showed reductions in brain inflammation [245]. That being said, there is need for ongoing research to elucidate the best delivery system and the optimal treatment duration with detailed analysis of any possible adverse effects.

Cancer is on the rise as a global threat, and the conventional tools we are utilizing are not the most effective. Therefore, “cocktail” approaches, including multicomponent delivery drugs and natural products, can be alternative nonclassical approaches. Polyphenols, including RSV, have been shown to act in synergy with classical drugs such as cisplatin, doxorubicin, paclitaxel, and 5-FU. In addition, derivatizing RSV remains an interesting option for enhancing its efficacy and delivery. For instance, the addition of methyl groups can increase the bio affinity of RSV, increasing its lipophilicity and improving its delivery to cells and tissues. Methylation and other derivatizing mechanisms can also protect the parent RSV from the detoxification machinery, which is considered a “xenobiotic”. As chemical derivatizing may remain costly, it would be interesting to challenge certain naturally modified systems (plants) to produce the desired derivative after genetic engineering. Considering the limitations of bioavailability and target specificity, the above-suggested derivatives and combination therapies can be loaded into nanoparticles, liposomes, nanocarriers, and micelles to overcome these limitations.

On the other hand, it is imperative to carefully characterize any off-target effects of RSV combinations, derivatives, or loaded therapeutics, including RSV. RSV, as a parent compound, has multiple molecular targets; RSV modifications can only add a plethora of targets that need to be characterized.

In addition to the biological efficacy still being extensively investigated for potentially effective modifications, as discussed in this report, the transition of RSV into clinical usage if furthered hurdled by several factors. Being a nutraceutical, various regulatory bodies impose different standards to ensure safety and compliance. The FDA issued, in the past, an advisory (FDA Advisory No. 2020-029) against purchase of some RESV supplements (PIPING ROCK Resveratrol Defense + C Dietary Supplement) for the lack of sufficient labelling and evaluation information. On the other side, the European Food Safety Authority (EFSA) concluded an intake of 150 mg/day for adults does not raise safety concerns [246].

## Figures and Tables

**Table 2:** Summary of the general RSV derivatives and their distinctive biological activities in different systems.

RSV Derivatives	Distinctive Biological Activities
Hydroxylated RSV Derivatives	Addition of hydroxyl groups to RSV molecules results in RSV derivatives that show the following [4,8,127]: Increased water solubility. Faster absorption. Greater bioavailability. Greater metabolic stability. Increased metabolic activity. Antioxidative, anti-inflammatory, anticancer, and immunomodulatory effects.
Aminated, Iminated and Amidated Derivative	Addition of amine groups to RSV molecules results in RSV derivatives that have the following properties [4,128]: Possess antioxidant activity. Possess moderate acetylcholinesterase inhibition. Show enhanced protection against glutamate excitotoxicity in neural cells (act as neuroprotectants).
Methoxylated Derivatives	Addition of methoxy groups to RSV molecules results in RSV derivatives that show the following [129,130,131]: Increased metabolic stability, bioavailability, and time needed to reach the peak plasma concentration. Increased lipophilicity. Improvement of oral absorption and cellular uptake. Low toxicity in animal and human models.
Prenylated Derivative	Prenylation of RSV molecules results in RSV derivatives that show the following [132,133]: Increased bioactivity. Promising results for the development of drugs. Increased ability to alter the blood–brain barrier.
Halogenated Derivatives	Addition of halogen groups to RSV molecules results in RSV derivatives that show the following [6]: Increased therapeutic potential. Greater bioavailability. Greater anticancer activity and more effective at suppressing tumor growth. Lower MICs against *C. albicans*.
Oligomerized Derivatives	RSV oligomerization results in RSV derivatives that show [8,134]: Increased biological effectiveness and specificity. Greater scavenging capacity.
Glycosylated Derivatives	Addition of glycosidic functional groups to RSV molecules results in RSV derivatives that show [4,6,135]: Increased water solubility. Increased bioavailability and less susceptibility to enzymatic oxidation. Enhanced oral absorption. Antioxidant and anti-inflammatory properties.

**Table 3:** Summary of the most relevant RSV nanocarrier systems and associated biological outcomes.

Nanocarrier System	Composition	Important Findings	Reference
Nanoemulsion-loaded thermosensitive hydrogel	Resveratrol-loaded coconut oil	⁻ The produced hydrogel of resveratrol nanoemulsion was cytotoxic to breast cancer cells. ⁻ Within 6 h, the in vitro release profile demonstrated a release rate of 80%.	[153]
Glycosylated liposomes	Resveratrol	⁻ RSV-galactosylated liposomes have strong antimicrobial properties against Gram-positive bacteria such as *Staphylococcus epidermidis* and *Methicillin resistant Staphylococcus aureus* at an RSV concentration of 10- to 60-fold under the minimum inhibitory concentration depending on the biofilm species.	[154]
Liposomes	Resveratrol	⁻ The expression levels of cancer-associated fibroblast markers, including α-SMA and IL-6, which typically support cancer cell proliferation and metastasis, were decreased in activated fibroblasts treated with RSV. ⁻ The activated fibroblasts enhanced the invasive properties and drug resistance of CRC cells in co-culture settings involving both 2D and 3D cultures, and this effect was counteracted by RSV treatment in the activated fibroblasts.	[155]
Polymeric micelles	Double-loaded doxorubicin/resveratrol	⁻ The release of doxorubicin depended on the pH of the medium and was faster than that of resveratrol. ⁻ The simultaneous delivery of doxorubicin and RSV via the micellar system enabled the cytotoxicity of doxorubicin in lymphoma cells and lowered its cardiotoxicity in cardiac cells.	[156]
Polymeric micelles	Co-delivery of quercetin/RSV and RSV/curcumin	⁻ Co-administration of micellar formulations containing resveratrol and curcumin with Adriamycin resulted in a notable decrease in ovarian tumor size, demonstrating their potential to alleviate Adriamycin-induced cardiotoxicity.	[157]
Lactobionic/Folate Dual-Targeted Amphiphilic Maltodextrin-Based micelles	Sulfasalazine (anticancer agent) and resveratrol	⁻ The dual-targeted micelles exhibited enhanced antitumor efficacy against hepatocellular carcinoma, along with increased cytotoxicity and internalization into HepG-2 liver cancer cells, decreased liver/body weight ratio, suppression of angiogenesis, and heightened apoptosis.	[158]
Solid Lipid Nanoparticles	D-α-Tocopheryl polyethylene glycol 1000 succinate–resveratrol	⁻ The RSV-loaded SLNs protected against the hydrolysis and oxidation of drugs and enhance bioavailability. ⁻ They also induced apoptosis in SKBR3/PR breast cancer cells and SKBR3/PR xenograft tumor models more efficiently than free RSV.	[159]
Gold nanoparticles	Resveratrol	⁻ By upregulating caspase-8 and Bax, and downregulating pro-caspase-9, pro-caspase-3, PI3K, and Akt, cell proliferation was reduced and apoptosis was increased in human hepatoma HepG2 cells.	[160]
PEG-nanoparticles coated with chitosan	Resveratrol	⁻ Increases bioavailability and reduces colon tumor growth compared to free RSV in xenograft and orthotopic implantation models of athymic mice.	[161]
Poly (lactic-co-glycolic acid) nanoparticles conjugated with lactoferrin	Resveratrol	⁻ The RSV-loaded NPs conjugated with lactoferrin, a natural iron-binding cationic glycoprotein that targets brain capillaries, helped them internalize into human brain microvascular endothelial cells forming the blood–brain barrier and accumulating in the brain as compared to unconjugated RSV-NPs and to free RSV.	[162]
Nanostructured lipid carriers	Lecithin and resveratrol	⁻ With the ability to remain stable at both room temperature and 4 °C for up to 12 months, this low-cost NLC possesses inherent anti-oxidant and anti-cancer properties.	[163]
